# Protein S Heerlen mutation heterozygosity is associated with venous thrombosis risk

**DOI:** 10.1038/srep45507

**Published:** 2017-04-04

**Authors:** P. Suchon, M. Germain, A. Delluc, D. Smadja, X. Jouven, B. Gyorgy, N. Saut, M. Ibrahim, J. F. Deleuze, M. C. Alessi, P. E. Morange, D. A. Trégouët

**Affiliations:** 1 Laboratory of Haematology, La Timone Hospital, Marseille, France; 2 Institut National pour la Santé et la Recherche Médicale (INSERM), Unité Mixte de Recherche en Santé(UMR_S) 1062, Nutrition Obesity and Risk of Thrombosis, Aix-Marseille University, Marseille, France; 3 Sorbonne Universités, UPMC Univ. Paris 06, INSERM, UMR_S 1166, Team Genomics & Pathophysiology of Cardiovascular Diseases, Paris, France; 4 ICAN Institute for Cardiometabolism and Nutrition, Paris, France; 5 Université de Brest, EA3878 and CIC1412, 29238 Brest, France; 6 Service d’hématologie biologique, AP-HP, Hôpital Européen Georges Pompidou, Paris, France; 7 Université Paris Descartes, Sorbonne Paris Cité, France, Inserm UMR-S1140, Paris, France; 8 INSERM, UMR-S970, Department of Epidemiology, Paris, France; 9 Université Paris Descartes, Sorbonne Paris Cité, Faculté de Médecine, Paris, France; 10 APHP, Georges Pompidou European Hospital, Department of Cardiology, Paris, France; 11 Centre National de Génotypage, Institut de Génomique, CEA, 91057 Evry, France; 12 CEPH, Fondation Jean Dausset, Paris, France

## Abstract

Hereditary Protein S (PS) deficiency is a rare coagulation disorder associated with an increased risk of venous thrombosis (VT). The PS Heerlen (PSH) mutation is a rare S501P mutation that was initially considered to be a neutral polymorphism. However, it has been later shown that PSH has a reduced half-life *in vivo* which may explain the association of PSH heterozygosity with mildly reduced levels of plasma free PS (FPS). Whether the risk of VT is increased in PSH carriers remains unknown. We analyzed the association of PSH (rs121918472 A/G) with VT in 4,173 VT patients and 5,970 healthy individuals from four independent case-control studies. Quantitative determination of FPS levels was performed in a subsample of 1257 VT patients. In the investigated populations, the AG genotype was associated with an increased VT risk of 6.57 [4.06–10.64] (p = 1.73 10^−14^). In VT patients in whom PS deficiency was excluded, plasma FPS levels were significantly lower in individuals with PSH when compared to those without [72 + 13 *vs* 91 + 21 UI/dL; p = 1.86 10^−6^, mean + SD for PSH carriers (n = 21) or controls (n = 1236) respectively]. We provide strong evidence that the rare PSH variant is associated with VT in unselected individuals.

Protein S (PS) is a potent anticoagulant protein that down regulates thrombin formation via two mechanisms^1^. It can stimulate the proteolytic inactivation of coagulation factors Va and VIIIa by activated protein C (APC), and can also enhance FXa inhibition by interacting with tissue factor pathway inhibitor (TFPI). Although free PS (FPS) is the most active PS form, recent reports indicate that the PS-C4BP complex also exhibits APC- and TFPI-cofactor activities^2^,^3^,^4^.

Hereditary PS deficiency is a rare coagulation disorder associated with an increased risk of venous thrombosis (VT)^5^. As for other deficiencies in natural anticoagulant inhibitors (antithrombin and protein C), PS deficiency is due to rare or private mutations within the *PROS1* gene (database available online: http://www.hgmd.cf.ac.uk). Among these, the rs121918472 variation—referred to as the PS Heerlen mutation—is detected at a high frequency in the general population (0.5%). PS Heerlen is a S501P amino acid change polymorphism that causes a loss of N-linked glycosylation at position 499^6^, and was initially considered a neutral polymorphism. However, it was later shown that 1) PS Heerlen has a reduced half-life *in vivo*^7^, 2) and that heterozygous and homozygous PS Heerlen genotypes were associated with mildly reduced or low levels of plasma FPS, respectively^8^. Moreover, PS Heerlen was found to increase thrombin generation in the presence of APC compared to wild type PS^9^. Thus whether VT risk is increased in PS Heerlen heterozygous subjects remains uncertain. Indeed, Bertina *et al*. found no differences in mutation prevalence in a panel of 96 uncharacterized normal individuals as compared to 1,182 Dutch patients with unexplained VT (0.52% vs 0.68% respectively)^6^. Apart from this study with poorly described patients, the impact of PS Heerlen on VT has only been studied in families either with PS deficiency or with other combined genetic or acquired risk factors. In these situations, PS Heerlen was thought to increase VT susceptibility^10^.

Although PS Heerlen is more frequent than any other *PROS1* mutation, it is still not considered a common variant. Consequently, previous genome-wide association studies (GWASs) which mainly interrogate common genetic variations (i.e. frequencies higher than 1%) were not able to identify the mutation^11^.

In the present study, we aimed to systematically determine if there was an association between PS Heerlen and VT by combining four French case-control DNA samples totaling 4,173 patients and 5,970 healthy individuals.

## Results

Genotype distributions of the rs121918472 variant within the four case-control studies are shown in Table 1. No homozygous carrier of the rare rs121918472-G allele was observed. When samples were combined, the AG genotype was more frequent in VT patients than in healthy controls, 2.2% vs 0.4% respectively, and was associated with an increased VT risk (Odds Ratio OR) of 6.57 [4.06-10.64] (p = 1.73 10^−14^). The association was significantly heterogeneous across studies (p < 10^−3^) due to strong associations in the EDITH cohort (2.4% vs 0.4%), the MARTHA12 cohort (2.5% vs 0.6%), the EOVT/MARTHA cohort (2.4% vs 0%) samples, but not in FARIVE study samples (1.1% vs 1.8%). Note that the observed AG genotype frequency was higher in FARIVE controls with hypertension (2.9%, n = 246) than in those without (0.9%, n = 343) in whom the frequency was similar to the other cohort controls. Excluding the FARIVE study from the combined analysis increased the significance of the association between rs121918472 and VT (OR = 14.58 [7.42–28.65], p = 7.66 10^−15^).

We also examined whether the rs121918472-G allele frequency varied in specific VT subgroups, such as in patients with provoked or unprovoked VT, patients with deep vein thrombosis or pulmonary embolism, and patients with F5 or F2 G20210A mutations. We did not observe preferential associations in any specific strata.

Interestingly, the rs121918472 variant was not identified as a VT-associated polymorphism in the two most recent VT GWAS projects by INVENT^11^ and 23 and Me consortia^12^. In the INVENT GWAS, the Heerlen mutation imputation quality was extremely low, r^2^ = 0.15^11^, which is far lower than the gold standard of r^2^ > 0.8 for imputation quality, and the increased risk associated with the imputed rs121918472-G allele was OR = 1.46 [0.28–7.58], p = 0.655. Because 865 MARTHA/EOVT participants examined in the current study also took part in the INVENT study, we were able to calculate the Spearman correlation between the true rs121918472 genotype as provided by Illumina Exome array results, and the imputed dose-derived from GWAS arrays. The resulting low correlation of ρ = 0.25 illustrated how imputation techniques are not reliable for inferring rare variants such as rs121918472.

**Figure 1:**
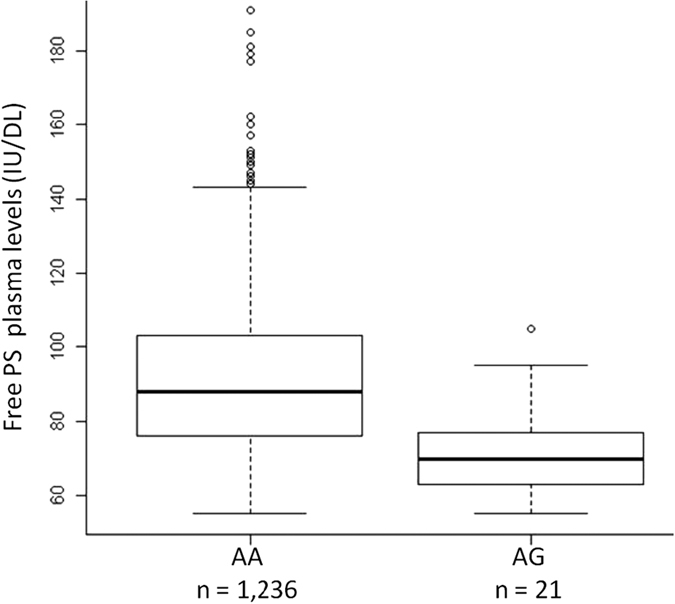
Free PS plasma levels (IU/dL) in MARTHA cases according to the rs121918472 polymorphism (age and sex adjusted P-value: 1.86 × 10^−6^).

To provide additional evidence implicating rs121918472 in increased VT risk, we assessed its association with plasma FPS levels in the MARTHA study samples. Even if by design FPS deficiency was excluded in this study, plasma FPS levels were significantly lower in individuals with PS Heerlen (p = 1.86 10^−6^) compared to those without (mean ± standard deviation: 72 ± 13 vs 91 ± 21 UI/dL, respectively) (Fig. 1).

## Discussion

In the present study we systematically analyzed four French case-control collections totalling 4,173 patients and 5,970 healthy individuals and provide strong evidence that the rare PS Heerlen variant is associated with VT.

In the investigated populations, the AG genotype was significantly more frequent in VT patients than in healthy controls, 2.2% vs 0.4%, respectively, and was also associated with an increased VT risk of 6.57. This association pattern was evident in EDITH, MARTHA12, and EOVT/MARTHA samples, but not in FARIVE study samples. There is no apparent explanation for the surprisingly high PS Heerlen mutation prevalence in apparently healthy individuals from the FARIVE study (1.8%). This observation could be due to the study design, as controls were actually matched cardiology patients without VT, and not healthy individuals from the general population. The significantly increased thrombosis risk associated with this mutation supports the concept that PS Heerlen has deleterious effects on the disease. However PS Heerlen is usually classified as a neutral polymorphism, both via bioinformatics prediction tools or by functional studies where it only slightly affects FPS levels in plasma. Of note, no homozygous carrier was identified in any of the four case-control studies. This observation was entirely compatible with data from the ExAC (Exome Aggregation Consortium) database, in which only one individual was homozygous for PS Heerlen among the ~33,000 studied subjects of European origin. This low frequency is likely due to the fact that homozygous carriers are considered to be protein S deficient, a condition with high morbidity. There is one PS Heerlen homozygous patient documented in the literature, with very low levels of free protein S (34%)^13^. In the MARTHA study, even if FPS deficiency was excluded by design, plasma FPS levels were significantly lower in individuals with PS Heerlen compared to those without. Assessing whether the disease effect is completely mediated through a direct effect on decreased FPS levels would require further investigations, including epidemiological studies where both the PSH mutation and FPS levels are measured in case-control samples. However, due to the extremely low frequency of the PSH variation in well matched control population, it will be nearly impossible to conduct mediation analysis to properly assess whether the effect of the mutation on disease risk is completely due to its effect on lowering FPS levels. However, in an additional population based study, only very low levels of FPS (<1/10^th^ percentile or <33 UI/dl) were associated with increased VT risk^1^,^14^, suggesting that the effect of PS Heerlen on VT risk might thus be related to mechanisms other than solely half-life reduction. However, recombinant PS Heerlen does not differ in either binding to, or dissociation to or from, immobilized C4BP *in vitro*, nor does it show any differences in the inactivation of FVa or FVIIIa^15^. PS is also a cofactor for TFPI, and accelerates the inhibition of activated factor X (FXa). The PS Heerlen mutation is located within the SHBG-like domain (Val243-Ser635) critical for TFPI binding and enhancement^16^. Thus, one might hypothesize that PS Heerlen could affect thrombosis risk by impairing TFPI function.

The recent GWAS on VT has enabled the identification of several new VT susceptibility variants. Very recently, the INVENT consortium gathered 7,507 VT subjects and 52,632 controls and identified two new loci (*SLC44A2* and *TSPAN15*) and robustly replicated six previously identified loci^11^. PS Heerlen was not among the identified disease polymorphisms, likely because this GWAS relied on imputation techniques that were not optimal for inferring rare variants^17^. The exome chip used in the present study was enriched by rare coding variants predicted to be causal. The results presented herein as well as those from the recently published paper on key hemostatic traits^18^ emphasize the promise of this new chip in identifying low-frequency variants associated with VT.

In conclusion, we provide evidence that PS Heerlen is associated with increased VT risk in individuals of the general population. Further investigations are required to better understand the surprisingly high prevalence of PS Heerlen mutation in apparently healthy individuals from the FARIVE study. Functional studies investigating the potential influence of PS Heerlen on VT risk would also be of benefit. These results also raise the question of whether PS Heerlen should always be genotyped irrespective of plasma PS levels in thrombophilia screening.

## Methods

The present work utilized four case-control sample cohorts (EDITH, FARIVE, EOVT/MARTHA)^11^ where VT events (pulmonary embolism or deep vein thrombosis) were objectively diagnosed^11^. Detailed descriptions of the four case-control sample groups are included in the supplementary text.

In the FARIVE and EDITH studies, the PS Heerlen variation was genotyped by High Resolution Melting (HRM). All EOVT and MARTHA patients were typed using the Illumina Human Exome Beadchip 12v1-2-A array and compared to control participants from the Paris Prospective Study^19^ typed with the Human Exome Beadchip 12v1-A chip (Illumina, Inc., Sand Diego, CA). Both of these genotyping arrays were designed to assess coding variants across the whole genome. Data analysis revealed 239,569 single nucleotide polymorphisms common to both arrays, including PS Heerlen rs121918472. Individuals with less than 98% genotyping success or high heterozygosity were excluded from the analyses, as were individuals demonstrating previously unknown close relatedness. The latter were separately assessed via pairwise clustering of identity by state distance (IBS) and multi-dimensional scaling (MDS) using PLINK software^20^. Genetic outlier analysis was performed using IBS statistics and principal component analysis to detect individuals of non-European origin, who were then removed from the analysis. This led to a selected cohort of 2,630 VT patients and 3,414 controls typed with Illumina exome chips for final association analysis.

The overall rs121918472 genotype call rate was 0.98, with 0.97 in the FARIVE and EDITH studies typed with HRM, and 0.99 in individuals typed with the Illumina Human Exome chips.

In MARTHA, fasting blood was drawn for FPS measurement. Quantitative FPS determination was performed by enzyme-linked immunosorbent assay (ELISA) using the Asserachrom FPS assay (Diagnostica Stago). PS deficiency was defined as FPS plasma antigen levels <55 IU/dL^−1^ using the specific ELISA assay. FPS data were available in 1,257 MARTHA individuals.

Genotype distribution of the rs121918472 was tested for deviation from Hardy-Weinberg (HW) equilibrium via the HW exact test. Association of rs121918472 with VT was analyzed via the Cochran-Armitage trend test and by logistic regression analysis adjusting for age and sex. Association of rs121918472 with FPS was assessed using linear regression analysis. Analyses were first performed separately in each study, and were then performed on all samples combined and further adjusted for the study group.

Informed consent was obtained from all participants, and the study met all institutional ethics requirement. The procedures employed were reviewed and approved by the *Assistance Publique des Hopitaux de Marseille* institutional review committee.

## Additional Information

**How to cite this article**: Suchon, P. *et al*. Protein S Heerlen mutation heterozygosity is associated with venous thrombosis risk. *Sci. Rep.* **7**, 45507; doi: 10.1038/srep45507 (2017).

**Publisher's note:** Springer Nature remains neutral with regard to jurisdictional claims in published maps and institutional affiliations.

## Supplementary Material

Supplementary Data

## Figures and Tables

**Table 1:** Association of the *PROS1* rs121918472 (PS Heerlen mutation) with VT in four French case-control sample studies.

	rs121918472	AG	GG	Cochran Armitage Trend P value	Age- and sex- adjusted Odds Ratio
	AA				
All					
Controls	5970 (99.6%)	22 (0.4%)	0	4.40 10^−18^	6.57 [4.06–10.64] p = 1.73 10^−14^
Cases	4173 (97.8%)	94 (2.2%)	0		
EDITH					
Controls	1156 (99.6%)	5 (0.4%)	0	7.54 10^−5^	5.59 [2.145–14.57] p = 4.29 10^−4^
Cases	1117 (97.6%)	27 (2.4%)	0		
FARIVE					
Controls	657 (98.2%)	12 (1.8%)	0	0.555	0.72 [0.30–1.72] p = 0.4565
Cases	706 (98.6%)	10 (1.4%)	0		
MARTHA12					
Controls	768 (99.4%)	5 (0.6%)	0	3.22 10^−3^	3.98 [1.48–10.72] p = 6.28 10^−3^
Cases	734 (97.5%)	19 (2.5%)	0		
EOVT/MARTHA					
Controls	3379 (100%)	0 (−)	0	8.52 10^−20^	Not applicable
Cases	1603 (97.6%)	40 (2.4%)*	0		

^*^The frequency of AG genotype was 2.7% and 2.4% in EOVT and MARTHA VT patients, respectively.
