# Supplementary material for: Protein S Heerlen mutation heterozygosity is associated with venous thrombosis risk
Source: Sci Rep. 2017 Apr 4;7:45507. doi: 10.1038/srep45507 (PMC5379621; doi:10.1038/srep45507)
Supplement: Supplementary Data [file srep45507-s1.docx]

Supplementary data

Protein S Heerlen mutation heterozygosity is associated with venous thrombosis risk

P Suchon^1,2^, M Germain^3,4^, A Delluc^5^, D Smadja^6^, X Jouven^7,8^, B. Gyorgy^3,4^_,_ N Saut^1^, M Ibrahim^1,2^, JF Deleuze^9,10^, MC Alessi^1,2^, PE Morange*^†1,2^, DA Trégouët^†3,4^

^1^Laboratory of Haematology, La Timone Hospital, Marseille, France; ^2^Institut National pour la Santé et la Recherche Médicale (INSERM), Unité Mixte de Recherche en Santé(UMR_S) 1062, Nutrition Obesity and Risk of Thrombosis, Marseille, France; Aix-Marseille University; ^3^Sorbonne Universités, UPMC Univ. Paris 06, INSERM, UMR_S 1166, Team Genomics & Pathophysiology of Cardiovascular Diseases, Paris, France; ^4^ICAN Institute for Cardiometabolism and Nutrition, Paris, France; ^5^Université de Brest, EA3878 and CIC1412, 29238 Brest, France ^6^Service d'hématologie biologique, AP-HP, Hôpital Européen Georges Pompidou, Paris, France, Université Paris Descartes, Sorbonne Paris Cité, France, Inserm UMR-S1140, Paris, France; ^7^INSERM, UMR-S970, Department of Epidemiology, Paris, France; Université Paris Descartes, Sorbonne Paris Cité, Faculté de Médecine, Paris, France; ^8^APHP, Georges Pompidou European Hospital, Department of Cardiology, Paris, France ; ^9^Centre National de Génotypage, Institut de Génomique, CEA, 91057 Evry,  France;^10^CEPH, Fondation Jean Dausset, Paris, France.

- EDITH [Oger E *et al* 2006]: The EDITH study is a case–control study that was designed to test interactions between genetic and environmental risk factors of VTE. Between May 2000 and December 2009, all consecutive unselected in-and outpatients seen at Brest (West of France) University Hospital for symptomatic VTE were asked to participate in the study. Controls were selected from the roster of patients hospitalized in the same ward in the following 12 months after the case’s event date. Controls were not included if they had a past history of VTE or if they were receiving long-term anticoagulant therapy.

- FARIVE [Tregouet DA *et al* 2009]: The FARIVE study is a multicenter case-control study of 607 patients with a first episode of proximal DVT and/or PE. Patients younger than 18 years, with previous VT event, that had a diagnosis of active cancer or a history of malignancy less than 5 years previously, or have a short life expectancy because of other causes, were excluded. The control group consists of age- and sex-matched individuals free of venous and arterial thrombotic disease. Potential control subjects with cancer, liver or kidney failure, or a history of venous and/or arterial thrombotic disease are ineligible.

- EOVT/MARTHA: For the third case-control collection, patients were selected from the EOVT and MARTHA Genome Wide Association Studies (GWAS) studies that have been extensively described in Tregouet DA *et al* 2009, Oudot-Mellakh T *et al* 2012, Antoni G *et al* 2010 and Germain M *et al* 2012. All patients were free of any chronic conditions and free of any well characterized genetic risk factors including anti-thrombin, protein C or protein S deficiency, homozygosity for FV Leiden or FII 20210A, and lupus anticoagulant. The control group was composed of 3,690 healthy subjects selected from the Paris Prospective Study 3 (PPS3) [Empana *et al* 2015].

- MARTHA12 [Germain *et al* 2015]: The MARTHA12 study is composed of an independent sample of 1,245 VT patients and 801 French healthy individuals. Patients have been recruited between 2010 and 2012 according to the same criteria as the MARTHA patients. The control group was composed of two subsamples of healthy individuals: one consists of 475 healthy subjects recruited from the Marseilles area, the second of 326 healthy heterozygous carriers of the FV Leiden or F2 G20210A mutations selected from the national health examination centers of the French Social Security in collaboration with the Hemostasis and Thrombosis Study Group.
